# How clinician-patient communication affects trust in health information sources: Temporal trends from a national cross-sectional survey

**DOI:** 10.1371/journal.pone.0247583

**Published:** 2021-02-25

**Authors:** Onur Asan, Zhongyuan Yu, Bradley H. Crotty

**Affiliations:** 1 School of Systems and Enterprises, Stevens Institute of Technology, Hoboken, New Jersey, United States of America; 2 Department of Medicine, Medical College of Wisconsin, Milwaukee, Wisconsin, United States of America; 3 Collaborative for Healthcare Delivery Science, Center for Advancing Population Science, Medical College of Wisconsin, Milwaukee, Wisconsin, United States of America; National Institutes of Health, UNITED STATES

## Abstract

**Background:**

Understanding patients’ trust in health information sources is critical to designing work systems in healthcare. Patient-centered communication during the visit might be a major factor in shaping patients’ trust in information sources.

**Objective:**

The purpose of this paper is to explore relationships between patient ratings of clinician communication during the visit and patient trust in health information sources.

**Methodology:**

We conducted a secondary analysis of the nationally-representative Health Information National Trends Surveys; HINTS4 Cycle1 (2011), HINTS4 Cycle4 (2014), and HINTS5 Cycle1 (2017), and HINTS5 Cycle2 (2018). We created a composite score of patient-centered communication from five questions and dichotomized at the median. We created multivariable logistic regression models to see how patient-centered communication influenced trust in different information sources across cycles. Consecutively, we used hierarchical analysis for aggregated data.

**Results:**

We analyzed data from 14,425 individuals. In the adjusted logistic models for each cycle and the hierarchical model, clinicians’ perceived patient-centered communication skills were significantly associated with increased trust in the clinicians as an information source.

**Conclusion:**

Clinicians still represent an essential source of trustworthy information reinforced by patient-centered communication skills. Given that trust helps build healing relationships that lead to better healthcare outcomes, communication sets an essential foundation to establish necessary trust. Interpreting information from the internet sources for patients is likely to remain a vital clinician function.

## 1. Introduction

Patient engagement is considered a core component of healthcare redesign and improvement [1, 2]. Understanding the communication and information exchange between patients and clinicians is a necessary precursor effectively engaging patients with their care [3, 4]. With the rapid advancement in communication technology broadly, new mediums for health information have been introduced. When patients have health-related questions, they turn to various old and new sources (friends, family, medical professionals, health websites, and social media) to fill gaps in their knowledge [5–8]. Trust is considered one of the primary factors influencing patients’ decision to choose and use various sources to access health-related information [9].

Clinicians have been the central and most respected source of information for the last decades, in part due to high levels of trust bestowed upon the profession [10, 11]. Trust is an essential component of any therapeutic relationship, defined as a patient’s expectation that his or her best interest will be kept in mind [12, 13]. Trust, at least in part, is mediated by patient-centered communication (PCC) [14–18], which has also been linked with treatment plan follow-through and clinical outcomes [19–24]. PCC is defined as a mutual understanding between clinicians and patients regarding patients’ health needs, values, and perspectives and sharing power and responsibility [25]. Studies have shown that patients’ perceptions of the quality of clinician communication in visits might have a more significant impact on outcomes than clinicians’ actual behaviors [26, 27]. Studies have also examined how different socioeconomic backgrounds influence trust in clinicians, hospitals, or healthcare systems [9]; findings are notable for African Americans having lower trust in their clinicians compared to white patients [28].

With the significant diffusion of technology into our lives, patients’ ability to access medical information has dramatically changed. Eighty percent of internet users in the U.S. have accessed health information using the internet [29], and a third of internet users have watched health-related videos on YouTube [30]. Technological advancements have also increased the number of channels for patients to get information from their clinicians, such as patient portals [31, 32], and mobile health applications [33].

In the current era of multiple information sources, it is not well-known what factors influence patients’ trust in information sources. Studies have explored patient trust in different information sources across demographics, such as age and ethnicity, and particular diseases like cancer [34, 35]. Other studies have also explored the impact of patient-clinician communication on both health outcomes and patient satisfaction using national databases [36–38]. Indeed, there is a growing body of literature on how the quality of clinician-patient communication might influence overall trust in clinicians [39, 40] and health outcomes [41]. However, the association between the perceived quality of clinician-patient communication in their in-person visits and patients’ trust in the information received from clinicians and other sources is still unclear. Larger data sets may shed light on this critical area. This study sought to determine whether there was a relationship between patient perceptions of communication with clinicians in visits and patient trust in medical information from different sources over time. We hypothesized that patients who experienced relatively good patient-centered communication with their clinicians during their visit might have high trust in clinicians, but lower trust in other medical information sources as compared to information from their clinicians. In more exploratory analyses, we sought to understand which aspects of PCC have more influence on trust in clinicians.

## 2. Methodology

### 2.1. Data

Data were derived from four cross-sectional surveys from the Health Information National Trends Survey: HINTS4 Cycle1 (2011), HINTS4 Cycle4 (2014), and HINTS5 Cycle1 (2017), and HINTS5 Cycle2 (2018). HINTS include U.S. residents who are 18 years and above, and the survey gathers information on U.S. resident’s requirements for access to and utilization of health-related data and health-related practices, insights, and awareness. There are 3,959 survey participants from HINTS4 Cycle1, 3,677 survey participants from HINTS4 Cycle4, 3,285 survey participants from HINTS5 Cycle 1, and 3,504 survey participants from HINTS5 Cycle2 studies separately. We used the weighted sample sizes to explore relationships between reported levels of patient-centered communication and trust in a variety of information sources. Information regarding the sampling design and survey procedures are available at http://hints.cancer.gov. (HINTS). This study does not involve patient participation, and no personal patient information has been revealed. All analysis was conducted using anonymized data which is publicly available, so the study does not require ethical approval IRB.

#### 2.1.1. Patient-centered communication

Patient-centered communication questions, derived from the HINTs surveys, have been used by various studies in different ways, including the creation of composite scores by converting questions to 0–100 scales [42], using the average score of communication questions to represent patient-centered communication [43], and developing a composite PCC score by summing all communication questions, which has been used previously [25, 44]. A composite of five questions created the patient-centered communication score to represent the additive effect among questions assessing communication between the participant and the clinician. Respondents answered the following questions regarding their interactions with their specific health care clinician they have seen within the last 12 months:

1. “How often did they give you the chance to ask all the health-related questions you had?”
2. “How often did they give the attention you needed to your feelings and emotions?”
3. “How often did they involve you in decisions about your health care as much as you wanted?
4. “How often did they make sure you understood the things you needed to do to take care of your health?”
5. “How often did they help you deal with feelings of uncertainty about your health or health care?”

Patients replied using a Likert scale, with options for the above questions being ‘*Always*,*’ ’Usually*,*’ ’Sometimes’ and ’Never*.’ We created a composite score for patient-centered communication by assigning values to individual item responses (‘*Always’ recorded as ‘3’*, *’Usually’ recorded as ‘2’*, *’Sometimes’ recorded as ‘1’ and ’Never’ recorded as ‘0’*) and summing to create a cumulative score range of 0–15. The median of this score was determined, which served as the cut point between high and low levels of patient-centered communication, dichotomizing high-quality communication as above the median and low-quality communication as below the median.

#### 2.1.2. Sources for medical information

The following questions were considered from HINTS4 Cycle1, HINTS4 Cycle4, and HINTS5 Cycle1, and HINTS5 Cycle2 surveys to evaluate participants’ willingness to trust medical information from different sources:

1. Doctor: “In general, how much would you trust information about health or medical topics from a doctor?”
2. Family: “In general, how much would you trust information about health or medical topics from family or friends?”
3. Radio: “In general, how much would you trust information about health or medical topics from the radio?”
4. internet: “In general, how much would you trust information about health or medical topics from the internet?”
5. Television: "In general, how much do you trust information about health or medical topics from Television?"
6. Newspaper: "In general, how much do you trust information about health or medical topics from Newspapers or magazines?"

The responses for the questions were: ’A lot,’ ’Some,’ ’A little,’ ’Not at All.’ We dichotomized responses by combining categories ’A lot,’ ’Some’ into ’High Trust’, and ’A little,’ ’Not at All’ into ’Low Trust.’ Notably, the medical information questions in HINTS Cycle 4–4 and 5–2 were in the context of cancer, whereas in HINTS Cycle 4–1 and 5–1, the questions represented a more general medical context.

#### 2.1.3. Covariates

We considered variables related to Age ("18–34","35–49","50–64","65–74","> = 75"), Race ("Hispanic", "Non-Hispanic White", "Non-Hispanic Black or African American", "Non-Hispanic Asian", "Non-Hispanic other"), Gender ("Male", "Female"), and Census region ("Northeast", "Midwest", "South", "West") as prespecified covariates of interest.

### 2.2. Statistical analysis

We followed similar methodologies as in prior studies when developing analytical plans published HINTS data [45–47]. We summarized the types of information sources (e.g. internet, television, clinician) and patient-centered communication (high and low levels) by sociodemographic factors. We employed a series of adjusted logistic models to ascertain the relationship between patient-centered communication and trust in information sources; we report both the random effect hierarchical model by year for all surveys and the individual models for the specific survey. We initially ran a series of 30 single logistic regression models, with a patient-centered communication score as the independent variable (low level as the reference for each model), and each of the six information sources as dependent variables for 4 HINTS cycles respectively. We then explored the relationship between trust in information from clinicians as the dependent variable, and each question in patient-centered communication as independent variables (chance to ask questions, feelings addressed, involved decisions, understood next steps, and help with uncertainty) in the multivariate logistic models for 4 HINTS cycles respectively.

To provide representative estimates of the U.S. population, we accounted for HINTS’ complex survey design using replicate weights, calculated using the Jack-Knife replication estimation method. We used chi-square tests to compare differences in proportions within each HINTS cycle by gender, age group, race/ ethnicity, and census region. We adjusted for sociodemographic and health-related factors, including gender, age group, race/ ethnicity, and census region. Multivariable logistic regression models were used to generate odds ratios (OR) and 95% confidence intervals (CI). We recorded the number of observations, null deviance, and residual deviance for all models. All analyses were completed using R statistical packages (mainly survey and lme4 package), and statistical significance was determined based on a p-value of 0.05.

## 3. Results

Using the sample size of 3,504 survey participants (estimated weighted sample size of 249,489,772) from HINTS5 Cycle2, more participants were female with 54.6% (weighted sample percentage 47.5%) and were between the age 50 and 64 years with 31.8% (weighted sample percentage 29.7%). Most of the sampled population were Non-Hispanic White race/ethnicity with 56.6% (weighted sample percentage 59.7%), and 43.4% belonged to the region of South (weighted sample percentage 37.7%). Those largest demographic groups in HINTS5 Cycle 2, as detailed above, are consistent with the other HINTS surveys included in this study (HINTS4 Cycle1, HINTS4 Cycle4, and HINTS5 Cycle1) with slight variation in numbers.

Generally, respondents reported having a high degree of trust in their doctors compared to other sources (Tables 1 and 2). All the demographic groups, including age, gender, region, and race groups, have more than 90% "high trust" in their doctors (95%) as an information source compared to the family (56%), internet (69%), radio (25%), news (42%) and television (37%). The average second highest trust group of information sources was the internet (with the age group of 75+ as an exception). We observed the least trust in radio as an information source.

**Table 1:** The percentages of high and low trust and patient-centered communication for each demographic group (age and gender) in HINTS5 Cycle2 (2018).

	*Hints 5 Cycle 2 (2018)*						
	Age					Gender	
	18–34	35–49	50–64	65–74	75+	Male	Female
**Trust**							
*Trust Doctor*							
High	376 (96.4%)	569 (94.7%)	909 (93.3%)	578 (93.7%)	367 (94.8%)	1157 (94.9%)	1642 (93.8%)
Low	12 (3.1%)	28 (4.7%)	45 (4.6%)	23 (3.7%)	14 (3.6%)	45 (3.7%)	77 (4.4%)
*Trust Family*							
High	225 (57.7%)	346 (57.6%)	547 (56.2%)	286 (46.4%)	192 (49.6%0	605 (49.6%)	991 (56.6%)
Low	161 (41.3%0	241 (40.1%)	392 (40.2%0	286 (46.4%)	154 (39.8%)	559 (45.9%)	675 (38.6%)
*Trust Radio*							
High	82 (21.0%)	157 26.1%)	233 (23.9%)	143 (23.2%0	75 (19.4%)	277 (22.7%)	413 (23.6%)
Low	301 (77.2%)	427 (71.0%)	697 (71.6%)	416 (67.4%)	265 (68.5%)	874 (71.7%)	1232 (70.4%)
*Trust Internet*							
High	259 (66.4%)	436 (72.5%)	694 (71.3%)	412 (66.8%)	186 (48.1%)	783 (64.2%)	1204 (68.8%)
Low	128 (32.8%)	150 (25.0%)	238 (24.4%)	165 (26.7%)	152 (39.3%)	377 (30.9%)	456 (26.1%)
*Trust TV*							
High	105 (26.9%)	200 (33.3%)	362 (37.2%)	228 (37.0%)	125 (32.3%)	404 (33.1%)	616 (35.2%)
Low	278 (71.3%)	384 (63.9%)	564 (57.9%)	339 (54.9%)	217 (56.1%)	747 (61.3%)	1035 (59.1%)
*Trust News*							
High	152 (39.0%)	266 (44.3%)	401 (41.2%)	241 (39.1%)	139 (35.9%)	451 (37.0%)	748 (42.7%)
Low	231 (59.2%)	317 (52.7%)	537 (55.1%)	321 (52.0%)	207 (53.5%)	701 (57.5%)	912 (52.1%)
**Patient Cantered Communication**							
*Chance to ask questions*							
High	273 (70.0%)	440 (73.2%)	751 (77.1%)	512 (83.0%)	320 (82.7%)	912 (74.8%)	1384 (79.1%)
Low	30 (7.7%)	39 (6.5%)	74 (7.6%)	43 (7.0%)	25 (6.5%)	79 (6.5%)	132 (7.5%)
*Feelings addressed*							
High	230 (59.0%)	383 (63.7%)	660 (67.8%)	459 (74.4%)	285 (73.6%)	791 (64.9%)	1226 (70.1%)
Low	73 (18.7)	96 (16.0%)	162 (16.6%)	98 (15.9%)	57 (14.7%)	195 (16.0%)	291 (16.6%)
*Involved decisions*							
High	255 (65.4%)	419 (69.7%)	707 (72.6%)	501 (81.2%)	301 (77.8%)	863 (70.8%)	1320 (75.4%)
Low							
*Understood next steps*							
High	273 (70.0%)	437 (72.7%)	762 (78.2%)	527 (85.4%)	309 (79.8%)	916 (75.1%)	1392 (79.5%)
Low	30 (7.7%)	42 (7.0%)	60 (6.2%)	29 (4.7%)	35 (9.0%)	71 (5.8%)	125 (7.1%)
*Help with uncertainty*							
High	220 (56.4%)	356 (59.2%)	644 (66.1%)	438 (71.0%)	265 (68.5%)	754 (61.9%)	1169 (66.8%)
Low	81 (20.8%)	123 (20.5%)	172 (17.7%)	114 (18.5%)	73 (18.9%)	226 (18.5%)	337 (19.3%)

**Table 2:** The percentages of high and low trust and patient-centered communication for each demographic group (region and race) in HINTS5 Cycle2.

	*Hints 5 Cycle 2 (2018)*								
	Region				Race				
	Northeast	Midwest	South	West	Non-Hispanic White	Non-Hispanic Black	Hispanic	Non-Hispanic Asian	Non-Hispanic Other
**Trust**									
*Trust Doctor*									
High	417 (94.6%)	527 (95.3%)	1190 993.9%)	665 (93.9%)	1798 (95.6%)	374 (91.2%)	397 (91.9%)	122 (94.6%)	108 (92.3%)
Low	15 (3.4%)	17 (3.1%)	58 (4.6%)	32 (4.5%)	65 (3.5%)	22 (5.4%)	26 (6.0%)	4 (3.1%)	5 (4.3%)
*Trust Family*									
High	228 (51.7%)	311 (56.2%)	701 (55.3%)	356 (50.3%)	1038 (55.2%)	217 (52.9%)	206 (47.7%)	63 (48.8%)	72 (61.5%)
Low	188 (42.6%)	216 (39.1%)	512 (40.4%)	318 (44.9%)	784 (41.7%)	158 (38.5%)	196 (45.4%)	56 (43.4%)	40 (34.2%)
*Trust Radio*									
High	103 (23.4%)	114 (20.6%)	312 (24.6%)	161 (22.7%)	367 (19.5%)	142 (34.6%)	117 (27.1%)	39 (30.2%)	25 (21.4%)
Low	308 (69.8%)	408 (73.8%)	886 (69.9%)	504 (71.2%)	1431 (76.1%)	232 (56.6%)	279 (64.6%)	78 (60.5%)	86 (73.5%)
Trust Internet									
High	274 (62.1%)	362 (65.5%)	866 (68.4%)	485 (68.5%)	1270 (67.5%)	280 (68.3%)	272 (63.0%)	90 (69.8%)	75 (64.1%)
Low	142 (32.2%)	163 (29.5%)	343 (27.1%)	185 (26.1%)	536 (28.5%)	101 (24.6%)	130 (30.1%)	28 (21.7%)	38 (32.5%)
*Trust TV*									
High	145 (32.9%)	161 (29.1%)	484 (38.2%)	230 (32.5%)	546 (29.0%)	207 (50.5%)	171 (39.6%)	57 (44.2%)	39 (33.3%)
Low	262 (59.4%)	365 (66.0%)	718 (56.7%)	437 (61.7%)	1252 (66.6%)	169 (41.2%)	227 (52.5%)	61 (47.3%)	73 (62.4%)
*Trust News*									
High	169 (38.3%)	210 (38.0%)	537 (42.4%)	283 (40.0%)	695 (36.9%)	205 (50.0%)	182 (42.1%)	60 (46.5%)	57 (48.7%)
Low	244 (55.3%)	319 (57.7%)	667 (52.6%)	383 (54.1%)	1113 (59.2%)	170 (41.5%)	217 (50.2%)	57 (44.2%)	56 (47.9%)
**Patient Centered Communication**									
*Chance to ask questions*									
High	344 (78.0%)	439 (79.4%)	973 976.4%)	540 (76.3%)	1530 (81.3%)	314 (76.6%)	274 (63.4%)	85 (65.9%)	93 (79.5%)
Low	37 (8.4%)	30 (5.4%)	92 (7.3%)	52 (7.3%)	117 (6.2%)	26 (6.3%)	48 (11.1%)	13 (10.1%)	7 (6.0%)
*Feelings addressed*									
High	308 (69.8%)	388 (70.2%)	865 (68.3%)	456 (64.4%)	1342 (71.3%)	282 (68.8%)	236 (54.6%)	70 (54.3%)	87 (74.4%)
Low	71 (16.1%)	80 (14.5%)	200 (15.8%)	135 (19.1%)	304 (16.2%)	57 (13.9%)	84 (19.4%)	28 (21.7%)	13 (11.1%)
*Involved decisions*									
High	334 (75.7%)	413 (74.7%)	941 (74.3%)	495 (69.9%)	1459 (77.6%)	296 (72.2%)	263 (60.9%)	78 (60.5%)	87 (74.4%)
Low	46 (10.4%)	55 (9.9%)	124 (9.8%)	94 (13.3%)	187 (9.9%)	42 (10.2%)	57 (13.2%)	20 (15.5%)	13 (11.1%0
*Understood next steps*									
High	354 (80.3%)	430 (77.8%)	994 (78.5%)	530 (74.9%)	1536 (81.7%)	310 (75.6%)	286 (66.2%)	85 (65.9%)	91 (77.8%)
Low	26 (5.9%)	38 (6.9%)	73 (5.8%)	59 (8.3%)	111 (5.9%)	28 (6.8%)	35 (8.1%)	13 (10.1%)	9 (7.7%)
*Help with uncertainty*									
High	290 (65.8%)	380 (68.7%)	818 (64.6%)	435 (61.4%)	1276 (67.8%)	268 (65.4%)	232 (53.7%)	72 (55.8%)	75 (64.1%)
Low	88 (20.0%)	85 (15.4%)	239 (18.9%)	151 (21.3%)	356 (18.9%)	69 (16.8%)	88 (20.4%)	26 (20.2%)	24 (20.5%)

Over half of the sampled population replied "Always" or "Usually" in all the patient-centered communication questions. More than 70% of patients from each demographics groups, including age, gender, region, and race (except Hispanic and Non-Hispanic Asian population) reported that they were given a chance to ask all the health-related questions they had (Variable: Chance to ask questions), and they understood the things they needed to do to take care of their health (Variable: Understood next steps). Slightly fewer patients, 65% on average, responded clinicians took into account their feelings and emotions (Variable: Feelings addressed), and that the clinician addressed feelings of uncertainty about health (Variable: Help with uncertainty).

Adjusted hierarchical and single logistic regression analyses are presented in Table 3. A summary of odds ratios and 95% confidence intervals are presented in Table 3. The detailed standard presentation of all regression model output for Tables 3 and 4 are added as S1 Appendix. In the single logistic analysis for each cycle, we identified significant relationships that included an association between higher levels of patient-centered communication and a higher level of trust in information from doctors (odds ratio > 2) across all four cycles. In one cycle (HINTS 5 Cycle 1, 2017), we found a significant association between higher levels of patient-centered communication and a higher level of trust in information from family/friends and the internet. We observed a significant association between PCC and trust in information from the internet only for general medical information; this was not significant for cancer information. Furthermore, aggregated data (hierarchical analysis) also confirmed our primary hypothesis, indicating a significant association between PCC and trust in information from clinicians. The hierarchical analysis also showed a significant association between PCC and trust in information from family and the internet.

**Table 3:** Odds ratios of aggregated patient-centered communication model predicting trusts, adjusted for race, gender, age, and geographic location.

	*Adjusted single logistic model*, *results are represented in OR (CI)*				
	Independent variable: Patient-Centered Communication				
	Hints All Cycles	Hints 4 Cycle 1 (2011)	Hints 4 Cycle 4 (2014)	Hints 5 Cycle 1 (2017)	Hints 5 Cycle 2 (2018)
	(both)	(medical)	(cancer)	(medical)	(cancer)
**Trust in information sources (dependent variable)**					
Trust information about health topics from a doctor	3.83***	2.687***	4.519***	5.760***	3.688**
	(2.82–5.20)	(1.723–4.189)	(2.543–8.032)	(2.679–12.384)	(1.703–7.985)
Trust about health topics from family or friends	1.24***	1.199	1.271	1.251*	0.866
	(1.12–1.36)	(0.941–1.528)	(0.979–1.651)	(1.037–1.510)	(0.650–1.154)
Trust on health topics from the radios	1.04	1.029	1.037	1.113	1.021
	(0.93–1.16)	(0.809–1.308)	(0.791–1.360)	(0.832–1.490)	(0.725–1.439)
Trust about medical topics from the internet	1.37***	1.308*	1.164	1.564**	1.024
	(1.23–1.53)	(1.045–1.638)	(0.833–1.628)	(1.190–2.054)	(0.756–1.387)
Trust information on medical topics from TV	1.1	1.286	1.048	1.009	1.157
	(0.99–1.21)	(0.998–1.657)	(0.795–1.381)	(0.785–1.297)	(0.876–1.528)
Trust information from newspaper or magazines	1.09	0.985	1.199	1.140	1.077
	(.994–1.20)	(0.803–1.208)	(0.900–1.597)	(0.882–1.473)	(0.810–1.431)

Note:

*p<0.05;

**p<0.01;

***p<0.001.

**Table 4:** Odds ratios of detailed patient-centered communication metrics in an adjusted model predicting willingness trust information from doctors.

	*Adjusted multivariate logistic model*, *results are represented in OR (CI)*				
	Dependent variable: How much would you trust information from a doctor?				
	Hints All Cycles	Hints 4 Cycle 1 (2011)	Hints 4 Cycle 4 (2014)	Hints 5 Cycle 1 (2017)	Hints 5 Cycle 2 (2018)
	(both)	(medical)	(cancer)	(medical)	(cancer)
**Patient-centered communication**					
Chance to ask questions	1.15	1.846	0.752	7.215*	0.381
	(0.80–1.66)	(0.860–3.960)	(0.257–2.198)	(1.511–34.455)	(0.110–1.315)
Feelings addressed	1.31	0.759	1.485	0.294^+^	3.659*
	(0.89–1.91)	(0.340–1.692)	(0.769–2.867)	(0.73–1.179)	(1.357–9.867)
Involved decisions	2.15***	2.107^+^	1.255	1.948	1.909
	(1.48–3.13)	(0.949–4.677)	(0.612–2.572)	(0.410–9.246)	(0.703–5.186)
Understood next steps	1.64*	3.102**	3.998***	0.717	0.626
	(1.12–2.40)	(1.626–5.918)	(2.076–7.701)	(0.163–3.154)	(0.203–1.937)
Help with uncertainty	1.47*	0.846	1.884	1.851	1.955^+^
	(1.04–2.10)	(0.418–1.713)	(0.830–4.277)	(0.562–6.100)	(1.009–3.791)

Note:

^+^p<0.1;

*p<0.05;

**p<0.01;

***p<0.001.

To further describe the relationship between trust in information from clinicians and each question in patient-centered communication, we used a multivariate logistic regression model for each cycle as well as a hierarchical model for aggregated data. Adjusted analyses are presented in Table 4. According to multivariate analysis for each cycle, patients having the chance to ask questions and understand the next steps (Variable: Chance to ask questions; and Understood the next steps) are more likely to trust information from clinicians for patients with general/all diagnoses. On the other hand, feelings or emotions being addressed by clinicians and understanding the next steps makes a difference in determining the levels of trust in clinicians from patients seeking cancer information (Variable: Feelings addressed and Understood next steps). Furthermore, a hierarchical model that eliminates differences across the years using aggregated data indicates that three specific PCC items, "Involved Decisions," "Understood next steps," and "Help with uncertainty," are the significant overall predictors of trust in information from clinicians.

## 4. Discussion

This study explored the association between patients’ perceived patient-centered communication scores and patients’ trust in health information sources using a nationally representative data set of U.S. households between 2011 and 2018. Our primary hypothesis—that patients who experienced relatively good patient-centered communication with their clinicians during their primary care visit might have high trust in clinicians—was supported by data within cycles as well as when combined in aggregate. This suggests that PCC is essential for building a trust relationship between patients and clinicians as the information source.

We identified several valuable insights that are important to consider in our changing healthcare landscape: (1) clinicians remain the most trusted information sources over time, with the internet rating second, higher than family/friends and traditional media; (2) patient-centered communication remains associated with higher trust in information from clinicians and the internet for medical, but not cancer-related, information; (3) we identified differences by age, but not other demographic factors, with respect to the use of information sources such as the internet [48]. Our findings suggest that even as patients access more information and services digitally, effective clincial communication and partnership [49] is a key to trust, and susbequent use, of information.

Overall, this study confirmed that clinicians remain the must trusted information sources over time. These results also show that these three sources are the major sources for information, with clinicians still being the primary ones. Due to the changes in our health care systems, including increased prevalence of long-term conditions, increased cost-of services, and high-deductible plans, self-management has become an essential paradigm in our current health care delivery model [50]. Patients are increasingly interested in solving their problems expeditiously, and increasingly this begins with an internet search rather than starting with a clinician. Therefore, internet sources are becoming so essential in seeking health information for better self-management, potentially impacting health care decisions and outcomes. Some studies also reported that information receiving from the internet might increase the feeling of patient empowerment [50].

Patients’ perceived quality of patient-centered communitation in the office visit was associated with higher trust in the information that clinicians provide. Further, the quality of communication is associated with higher trust in internet sources for medical topics, but not cancer. Aggregation of all cycles with hierarchical adjusted analysis showed the perception of patient-centered communication in the visit has a significant association with trust in information from clinicians, the internet, and families and friends, with the caveat that the internet and family/friends this association was not seen across all time points. Taken together, these findings suggest that clinician communication might modulate trust in information from other sources.

Specific components of communication, including shared decision making and active planning, shape trust in information provided by clinicians. The aggregated hierarchical analysis showed that trust in information from clinicians is significantly influenced by three specific parameters of patient-centered care "involved decisions," "understood next steps," and "help with uncertainty." On the other hand, the single regression models for each cycle also revealed interesting results. "Understood next steps" and "Feeling addressed" were the most important aspect of patient-centered communication influencing trust in information from a clinician for the context of cancer. For the general medical context, the most significant items were "Understood next step" and "Chance to ask questions." Establishing trust with the patients is critical for every clinician to have a long and effective relationship with their patients. The findings of this study imply that clinicians need to make sure patients understand all of the next steps when they leave the visit to establish higher trust. Nowadays, “after visit summaries” might be one efficient way to enforce patients’ understanding of the next steps. One study showed that some clinicians prepare after-visit summary with patients during the visit by sharing the screen with them to enforce their understanding [51], which can promote trust [52]. Encouraging patients to access online records, including notes, which extends and promotes further relayance of information even after a visit, may help reinforce understanding in general [53, 54], as well as in cancer-related contexts [55].

Clinicians who are effetive communicators may help patients navigate internet sources, and it is plausible that patients who experience better communication develop a better skill set navigating the broad, heterogeneous internet sources. Search engines can juxtapose professional content and anecdotal experiences, as well as unfounded or biased content, leaving patients to sift through content. Patients may need help from clinicians applying this information to individual circumstances and cases. Another study using the HINTS database from the 2007 cycle also showed that when patients perceive less patient-centered communication from their clinicians, they were more likely to seeking health information online [56]. We did not observe people shifting to the internet or trusting it more as a substitute for clinicians, even if the perceived communication with clinicians is poor. Intuitively, it makes sense for people to turn to other sources for information when they perceive they are not receiving effective communication from their clinicians. Patients, however, may be better served by having effective partnerships to navigate such online information.

Notably, our association of PCC with trust in internet did not extend to cancer-specific content. This might indicate that patients have substantial trust in a clinician when it comes to critical information such as cancer. Conversely, the internet may be more ‘noisy’ for more serious diseases. This finding is important as patients–and their at-home caregivers–increasingly become engaged in self-management in serious illness. For example, internet can help with filling in knowledge gaps, or for preparing for visits with clinicians [57–59]. Patients need to be able to trust that the information helping them guide their decisions is reputable, accurate, and useful. While professional information may be more broad, social media enables people to share their more personal experiences with medications, treatments, and experiences. Unfortunately, this content is unlikely to apply to all readers, and research has identified approximately 20% of cancer-related social media information is not medically or scientifically accurate [59] and reliability is low [60]. Accessing reliable information in a timely manner is one of the critical aspects of today’s health care, given the great emphasis on the patient-centered care model.

Descriptive statistics of high and low trust across a range of patient characteristics indicate that all groups have high overall trust in health topics from their clinicians. Trust in health topics from the internet ranged between 48% to 72%, with 65 years + group having the lowest trust in health topics from the internet according to the most recent cycle (2018). Due to an increase in the aging population, self-management and skills to navigate health-related might be more critical among this population [61]. Whites, African Americans, and Hispanics have the relatively same percentage of the population having high trust in health topics from clinicians as well as the internet, different from the previous studies reported lower trust for African Americans [28, 62]. The percentage of high trust in health topics from families and friends was also similar across ethnicity groups.

Taken together, our findings suggest that effective clincial communication and partnership is associated with trust, often a prerequisite to using and acting upon information for health-realated purposes given that more informed patients participate more in their care management [63], and they have better follow through with care plans [64]. It remains even more important to have trusted advisors as time with clinicians decreases and people become accustomed to ‘online research’ to gather information or appraise decisions in multiple aspects of their lives. Google, for example, receives more than 1 billion health questions every day. As described above, the content of this information is quite heterogeneous. Ethical obligations to help patients develop appraisal skills and promote reputable information have been raised [65]. Given that, part of the clinicians’ role will help patients navigate information from the internet, as opposed to telling patients not to search their conditions as was done in the past [66]. High-quality communication with their clinicians helps patients understand the information they consume better from any sources, including the internet.

This study has limitations that must be taken into consideration. While the HINTS questions focused on in-visit communication, further research is needed to explore the quality and type of information exchanged between patients and clinicians online and/or beyond the visit and those impacts on outcomes such as trust. It is important to note that patient-clinician communication is no longer confined to the walls of the office, and itself is increasingly taking place online through secure messaging [67–70] and telehealth [71]. While a strength of the study was that it followed a set of standard and well-studied survey questions, the questions did not allow more detailed descriptions of information, such as distinguishing different types of information. The internet is a medium of heterogeneous sources. However, many patients may have difficulty navigating reputable and nonreputable sources that may be juxtaposed next to one another. With many patients starting information in search engines, it is reasonable to consider en masse how patients trust that process. Lastly, association does not mean causation.

## 5. Conclusion

Our study provides evidence that patients, regardless of their demographic differences, have more trust in health topics received from their clinicians compared to other sources if they perceive patient-centered communication to be relatively high in their visits. The rate of using consumer information technologies to access health information may turn clinicians into facilitators and mediators between patients and online health information sources. Clinicians can also play an essential role in increasing the adoption and use of information technologies among patients. Finally, new information technologies should be designed to facilitate information transfer between clinicians and patients in a user-centered way.

## Supporting information

S1 Appendix (PDF)
