# Supplementary material for: How clinician-patient communication affects trust in health information sources: Temporal trends from a national cross-sectional survey
Source: PLoS One. 2021 Feb 25;16(2):e0247583. doi: 10.1371/journal.pone.0247583 (PMC7906335; doi:10.1371/journal.pone.0247583)
Supplement: S1 Appendix — (PDF) [file pone.0247583.s001.pdf]

	Dependent variable:			
	Trust information about health topics from a doctor			
	Hints 4 Cycle 1 (2011)	Hints 4 Cycle 4 (2014)	Hints 5 Cycle 1 (2017)	Hints 5 Cycle 2 (2018)
Patient Centered Communication	2.687***	4.519***	5.760***	3.688**
	(1.723−4.189)	(2.543−8.032)	(2.679−12.384)	(1.703−7.985)
Region: Midwest	.920	.768	.322*	.442
	(.391−2.164)	(.313−1.886)	(.114−.910)	(.116−1.680)
Region: South	.453*	.653	.527	.482+
	(.225−.912)	(.299−1.428)	(.192−1.446)	(.231−1.006)
Region: West	.418+	.854	.551	.772
	(.165−1.058)	(.411−1.776)	(.214−1.423)	(.226−2.641)
Hispanic	.587	.527	.477	.407
	(.230−1.495)	(.248−1.120)	(.201−1.135)	(.100−1.665)
Non-Hispanic Black or African American	1.287	.496	.522	.557
	(.641−2.587)	(.153−1.608)	(.188−1.455)	(.271−1.144)
Non-Hispanic Other	.949	.439	.349+	.986
	(.356−2.528)	(.106−1.829)	(.104−1.165)	(.190−5.106)
Hispanic Asian	1.246	.622	1.891	3.336
	(.395−3.933)	(.191−2.026)	(.117−30.527)	(.354−31.462)
Age: 35-49	.536	.830	.323	.449
	(.205−1.401)	(.367−1.877)	(.073−1.436)	(.103−1.962)
Age: 50-64	.488+	.927	.284*	.460
	(.228−1.045)	(.456−1.886)	(.085−.952)	(.146−1.447)
Age: 65-74	.593	.792	.198*	.543
	(.286−1.230)	(.368−1.703)	(.049−.807)	(.173−1.697)
Age: 75+	.464+	.985	.317	.562
	(.200−1.075)	(.421−2.308)	(.045−2.243)	(.166−1.909)
Female	1.141	1.521	1.011	.876
	(.667−1.952)	(.926−2.499)	(.515−1.982)	(.444−1.731)
Constant	43.833***	17.758***	121.290***	80.332***
	(16.477−116.604)	(8.235−38.294)	(27.657−531.911)	(29.195−221.037)
Observations	3,587	3,039	2,726	2,921
Residual Deviance	1,350	1,284	805	878
Null Deviance	1,420	1,369	885	946
Note:				+p<0.1; *p<0.05; **p<0.01; ***p<0.001

	Dependent variable:			
	Trust about health topics fram family or friends			
	Hints 4 Cycle 1 (2011)	Hints 4 Cycle 4 (2014)	Hints 5 Cycle 1 (2017)	Hints 5 Cycle 2 (2018)
Patient Centered Communication	1.199	1.271^{+}	1.251*	.866
	(.941−1.528)	(.979−1.651)	(1.037−1.510)	(.650−1.154)
Region: Midwest	.726^{+}	.976	.688^{+}	.863
	(.516−1.021)	(.663−1.436)	(.462−1.023)	(.580−1.284)
Region: South	.857	.879	.736	.898
	(.604−1.214)	(.591−1.307)	(.514−1.054)	(.646−1.248)
Region: West	.836	1.080	.684^{+}	.870
	(.593−1.178)	(.747−1.561)	(.469−.997)	(.602−1.256)
Hispanic	.579**	.514**	.936	.808
	(.403−.831)	(.354−.745)	(.600−1.458)	(.569−1.149)
Non-Hispanic Black or African American	.829	1.264	.722^{+}	1.339
	(.537−1.278)	(.790−2.023)	(.508−1.025)	(.900−1.993)
Non-Hispanic Other	1.172	1.108	.980	1.023
	(.677−2.026)	(.538−2.285)	(.501−1.916)	(.483−2.167)
Hispanic Asian	.840	1.696	1.679^{+}	1.125
	(.470−1.502)	(.876−3.284)	(.986−2.859)	(.526−2.405)
Age: 35-49	.784	.926	.738^{+}	1.161
	(.543−1.131)	(.621−1.381)	(.539−1.012)	(.758−1.778)
Age: 50-64	.601**	.881	.856	1.002
	(.436−.829)	(.614−1.265)	(.612−1.197)	(.655−1.532)
Age: 65-74	.433***	.662^{+}	.707^{+}	.666^{+}
	(.296−.635)	(.438−.999)	(.474−1.056)	(.423−1.048)
Age: 75+	.530**	.612*	.606*	.976
	(.351−.800)	(.390−.959)	(.403−.911)	(.636−1.500)
Female	1.152	1.280^{+}	1.073	1.411*
	(.902−1.470)	(.987−1.659)	(.804−1.431)	(1.079−1.844)
Constant	2.418***	1.384	1.885**	1.360
	(1.554−3.763)	(.830−2.309)	(1.226−2.899)	(.854−2.166)
Observations	3,511	2,930	2,691	2,830
Residual Deviance	4,647	3,945	3,655	3,864
Null Deviance	4,730	4,036	3,704	3,916
Note:				$^{+}$p<0.1; *p<0.05; **p<0.01; ***p<0.001

	Dependent variable:			
	Trust on health topics from the radios			
	Hints 4 Cycle 1 (2011)	Hints 4 Cycle 4 (2014)	Hints 5 Cycle 1 (2017)	Hints 5 Cycle 2 (2018)
Patient Centered Communication	1.029	1.037	1.113	1.021
	(.941−1.528)	(.979−1.651)	(1.037−1.510)	(.650−1.154)
Region: Midwest	.636*	1.063	.749	1.184
	(.516−1.021)	(.663−1.436)	(.462−1.023)	(.580−1.284)
Region: South	.863	.834	.880	1.083
	(.604−1.214)	(.591−1.307)	(.514−1.054)	(.646−1.248)
Region: West	.749	.863	.643^{+}	1.016
	(.593−1.178)	(.747−1.561)	(.469−.997)	(.602−1.256)
Hispanic	2.028***	1.694*	1.265	1.846**
	(.403−.831)	(.354−.745)	(.600−1.458)	(.569−1.149)
Non-Hispanic Black or African American	1.728*	1.778**	1.217	3.575***
	(.537−1.278)	(.790−2.023)	(.508−1.025)	(.900−1.993)
Non-Hispanic Other	1.493	.711	1.603	.882
	(.677−2.026)	(.538−2.285)	(.501−1.916)	(.483−2.167)
Hispanic Asian	2.414**	1.794	2.120*	2.054^{+}
	(.470−1.502)	(.876−3.284)	(.986−2.859)	(.526−2.405)
Age: 35-49	.695^{+}	1.403	1.140	1.033
	(.543−1.131)	(.621−1.381)	(.539−1.012)	(.758−1.778)
Age: 50-64	1.000	1.329	1.510	1.015
	(.436−.829)	(.614−1.265)	(.612−1.197)	(.655−1.532)
Age: 65-74	.686^{+}	1.198	1.072	1.115
	(.296−.635)	(.438−.999)	(.474−1.056)	(.423−1.048)
Age: 75+	.667^{+}	1.127	1.218	1.068
	(.351−.800)	(.390−.959)	(.403−.911)	(.636−1.500)
Female	.852	1.068	1.036	1.112
	(.902−1.470)	(.987−1.659)	(.804−1.431)	(1.079−1.844)
Constant	.581*	.255***	.279***	.203***
	(1.554−3.763)	(.830−2.309)	(1.226−2.899)	(.854−2.166)
Observations	3,441	2,893	2,665	2,796
Residual Deviance	4,207	3,313	2,937	3,058
Null Deviance	4,339	3,364	2,979	3,171
Note:				$^{+}$p<0.1; *p<0.05; **p<0.01; ***p<0.001

	Dependent variable:			
	Trust about medical topics from the internet			
	Hints 4 Cycle 1 (2011)	Hints 4 Cycle 4 (2014)	Hints 5 Cycle 1 (2017)	Hints 5 Cycle 2 (2018)
Patient Centered Communication	1.308*	1.164	1.564**	1.024
	(1.045−1.638)	(.833−1.628)	(1.190−2.054)	(.756−1.387)
Region: Midwest	1.001	.992	.661*	1.216
	(.655−1.529)	(.614−1.603)	(.461−.948)	(.805−1.839)
Region: South	.815	.968	.617*	1.416*
	(.574−1.157)	(.627−1.493)	(.422−.904)	(1.030−1.947)
Region: West	.962	1.206	.689^{+}	1.410
	(.655−1.414)	(.776−1.874)	(.466−1.018)	(.888−2.238)
Hispanic	1.134	.875	.984	.876
	(.770−1.668)	(.582−1.315)	(.622−1.558)	(.634−1.210)
Non-Hispanic Black or African American	.656	1.663	.793	1.001
	(.401−1.073)	(.893−3.095)	(.529−1.188)	(.667−1.502)
Non-Hispanic Other	.530^{+}	.686	.810	.608
	(.256−1.097)	(.314−1.496)	(.389−1.687)	(.256−1.441)
Hispanic Asian	1.911*	1.133	2.140*	1.639
	(1.059−3.449)	(.425−3.026)	(1.085−4.221)	(.765−3.509)
Age: 35-49	.861	1.350	.941	1.261
	(.540−1.375)	(.895−2.035)	(.562−1.576)	(.735−2.161)
Age: 50-64	1.083	1.411*	1.027	1.333
	(.682−1.721)	(1.027−1.939)	(.688−1.535)	(.826−2.149)
Age: 65-74	.504**	.893	.686*	1.286
	(.328−.774)	(.623−1.279)	(.493−.955)	(.794−2.082)
Age: 75+	.221***	.695	.329***	.577*
	(.132−.369)	(.432−1.120)	(.194−.560)	(.357−.932)
Female	1.319*	1.612***	1.235	1.433*
	(1.020−1.707)	(1.258−2.066)	(.950−1.604)	(1.095−1.876)
Constant	3.464***	1.584*	3.067***	1.260
	(2.119−5.663)	(1.030−2.435)	(2.023−4.648)	(.701−2.262)
Observations	3,424	2,910	2,671	2,820
Residual Deviance	3,651	3,430	3,130	3,496
Null Deviance	3,815	3,511	3,235	3,561
Note:				$^{+}$p<0.1; *p<0.05; **p<0.01; ***p<0.001

	Dependent variable:			
	Trust information on medical topics from TV			
	Hints 4 Cycle 1 (2011)	Hints 4 Cycle 4 (2014)	Hints 5 Cycle 1 (2017)	Hints 5 Cycle 2 (2018)
Patient Centered Communication	1.286^{+}	1.048	1.009	1.157
	(.998−1.657)	(.795−1.381)	(.785−1.297)	(.876−1.528)
Region: Midwest	.787	1.027	.686	.856
	(.582−1.064)	(.654−1.611)	(.438−1.074)	(.524−1.398)
Region: South	.775	.766	.816	1.246
	(.566−1.061)	(.516−1.137)	(.599−1.111)	(.910−1.707)
Region: West	$.667^{*}$	.698	$.588^{**}$	.953
	(.471−.945)	(.443−1.099)	(.415−.832)	(.638−1.422)
Hispanic	2.219^{***}	3.007^{***}	1.510^{+}	2.193^{***}
	(1.597−3.084)	(1.967−4.598)	(.980−2.325)	(1.469−3.274)
Non-Hispanic Black or African American	2.429^{***}	3.351^{***}	1.974^{**}	3.101^{***}
	(1.614−3.655)	(2.103−5.339)	(1.280−3.047)	(2.153−4.466)
Non-Hispanic Other	1.296	.688	1.442	1.294
	(.672−2.500)	(.287−1.651)	(.654−3.183)	(.484−3.457)
Hispanic Asian	3.033^{**}	1.929^{+}	2.574^{**}	3.202^{*}
	(1.567−5.869)	(.915−4.068)	(1.392−4.759)	(1.362−7.529)
Age: 35-49	1.021	1.249	1.510	1.313
	(.714−1.460)	(.836−1.866)	(.894−2.550)	(.866−1.990)
Age: 50-64	1.145	1.294	1.777^{*}	1.864^{**}
	(.833−1.573)	(.876−1.910)	(1.133−2.789)	(1.221−2.846)
Age: 65-74	.865	1.353	1.722^{+}	1.959^{***}
	(.610−1.228)	(.907−2.019)	(1.011−2.935)	(1.392−2.758)
Age: 75+	$.671^{*}$	1.340	1.860^{+}	2.137^{**}
	(.470−.960)	(.842−2.132)	(.966−3.583)	(1.325−3.447)
Female	1.084	1.036	1.119	1.099
	(.849−1.384)	(.794−1.351)	(.847−1.479)	(.893−1.354)
Constant	$.615^{*}$	$.426^{**}$	$.347^{**}$	$.206^{***}$
	(.414−.912)	(.250−.726)	(.182−.662)	(.135−.312)
Observations	3,480	2,922	2,677	2,802
Residual Deviance	4,593	3,747	3,318	3,404
Null Deviance	4,761	3,930	3,416	3,594
Note:				$^{+}$p<0.1; *p<0.05; **p<0.01; ***p<0.001

	Dependent variable:			
	Trust information from newspapers or magazines			
	Hints 4 Cycle 1 (2011)	Hints 4 Cycle 4 (2014)	Hints 5 Cycle 1 (2017)	Hints 5 Cycle 2 (2018)
Patient Centered Communication	.985	1.199	1.140	1.077
	(.803−1.208)	(.900−1.597)	(.882−1.473)	(.810−1.431)
Region: Midwest	.716^{+}	1.285	.619^{+}	1.288
	(.507−1.011)	(.870−1.898)	(.381−1.005)	(.802−2.068)
Region: South	.856	1.119	.732	1.192
	(.643−1.139)	(.801−1.564)	(.465−1.151)	(.907−1.568)
Region: West	.728^{+}	1.022	.628*	1.156
	(.521−1.018)	(.634−1.646)	(.409−.964)	(.762−1.755)
Hispanic	1.396^{+}	1.180	1.279	1.117
	(.952−2.046)	(.761−1.829)	(.828−1.976)	(.773−1.614)
Non-Hispanic Black or African American	1.029	2.162***	1.198	2.383***
	(.693−1.529)	(1.479−3.160)	(.776−1.848)	(1.637−3.469)
Non-Hispanic Other	1.426	.604	.647	.850
	(.734−2.773)	(.303−1.205)	(.309−1.353)	(.454−1.589)
Hispanic Asian	1.626	2.673**	2.055*	1.760
	(.921−2.869)	(1.388−5.146)	(1.157−3.653)	(.833−3.718)
Age: 35-49	.852	1.123	1.228	1.197
	(.603−1.204)	(.775−1.629)	(.829−1.819)	(.786−1.821)
Age: 50-64	1.012	1.012	1.351^{+}	1.039
	(.703−1.457)	(.732−1.398)	(.981−1.859)	(.660−1.638)
Age: 65-74	.682*	.924	1.005	.938
	(.473−.983)	(.659−1.295)	(.693−1.456)	(.585−1.504)
Age: 75+	.702^{+}	.865	1.056	1.227
	(.481−1.024)	(.594−1.260)	(.613−1.817)	(.732−2.057)
Female	1.220^{+}	1.288*	1.240	1.263
	(.972−1.531)	(1.011−1.640)	(.965−1.593)	(.953−1.676)
Constant	.985	.525**	.718	.411**
	(.631−1.538)	(.341−.808)	(.430−1.199)	(.232−.727)
Observations	3,486	2,918	2,685	2,812
Residual Deviance	4,782	3,988	3,614	3,764
Null Deviance	4,837	4,084	3,677	3,839
Note:				$^{+}$p<0.1; *p<0.05; **p<0.01; ***p<0.001

	Dependent variable:			
	How much would you trust information from a doctor?			
	Hints 4 Cycle 1 (2011)	Hints 4 Cycle 4 (2014)	Hints 5 Cycle 1 (2017)	Hints 5 Cycle 2 (2018)
Chance to ask questions	1.846	.752	7.215*	.381
	(.860−3.960)	(.257−2.198)	(1.511−34.455)	(.110−1.315)
Feelings addressed	.759	1.485	.294+	3.659*
	(.340−1.692)	(.769−2.867)	(.073−1.179)	(1.357−9.867)
Involved decisions	2.107+	1.255	1.948	1.909
	(.949−4.677)	(.612−2.572)	(.410−9.246)	(.703−5.186)
Understood next steps	3.102**	3.998***	.717	.626
	(1.626−5.918)	(2.076−7.701)	(.163−3.154)	(.203−1.937)
Help with uncertainty	.846	1.884	1.851	1.955+
	(.418−1.713)	(.830−4.277)	(.562−6.100)	(1.009−3.791)
Region: Midwest	1.104	.629	.592	1.157
	(.357−3.419)	(.208−1.902)	(.207−1.695)	(.357−3.748)
Region: South	.476+	.746	.449	.663
	(.202−1.118)	(.301−1.849)	(.155−1.302)	(.285−1.542)
Region: West	.668	.647	.663	.983
	(.275−1.623)	(.276−1.518)	(.248−1.773)	(.279−3.462)
Hispanic	.670	.736	.387+	.611
	(.302−1.489)	(.304−1.782)	(.135−1.107)	(.175−2.128)
Non-Hispanic Black or African American	1.641	1.313	.858	.631
	(.785−3.427)	(.427−4.039)	(.172−4.289)	(.250−1.592)
Non-Hispanic Other	1.777	.835	.247*	.913
	(.399−7.919)	(.147−4.733)	(.069−.889)	(.129−6.443)
Hispanic Asian	.795	.334	5.161	4.872
	(.226−2.795)	(.076−1.466)	(.538−49.503)	(-)
Age: 35-49	.653	.413	.045**	1.287
	(.237−1.800)	(.134−1.275)	(.008−.242)	(.392−4.224)
Age: 50-64	.378*	.537	.039***	.650
	(.156−.915)	(.184−1.566)	(.009−.165)	(.169−2.491)
Age: 65-74	.412*	.456	.024***	1.514
	(.187−.906)	(.128−1.617)	(.005−.114)	(.389−5.889)
Age: 75+	.399	.562	.044**	1.009
	(.136−1.173)	(.154−2.045)	(.006−.311)	(.220−4.637)
Female	.838	.890	.671	.730
	(.491−1.432)	(.526−1.508)	(.334−1.347)	(.377−1.413)
Constant	15.771***	12.496***	488.802***	31.151***
	(5.236−47.503)	(3.697−42.241)	(94.188−2,536.699)	(8.258−117.510)
Observations	2,940	2,509	2,302	2,439
Residual Deviance	785	705	506	565
Null Deviance	877	807	603	642
Note:				+p<0.1; *p<0.05; **p<0.01; ***p<0.001
